# Exercise training during pregnancy reduces circulating insulin levels in overweight/obese women postpartum: secondary analysis of a randomised controlled trial (the ETIP trial)

**DOI:** 10.1186/s12884-017-1653-5

**Published:** 2018-01-08

**Authors:** Kirsti K. Garnæs, Siv Mørkved, Kjell Å. Salvesen, Øyvind Salvesen, Trine Moholdt

**Affiliations:** 1 Department of Circulation and Medical Imaging, NTNU, Norwegian University of Science and Technology, Box 8905, 7491 Trondheim, Norway; 2 Department of Public Health and General Practice, NTNU, Norwegian University of Science and Technology, Trondheim, Norway; 3 Research Department, St. Olavs Hospital Trondheim University Hospital, Trondheim, Norway; 4 Institute of clinical and molecular medicine, Norwegian University of Science and Tecnology, Trondheim, Norway; 5 Department of Obstetrics and Gynaecology, St. Olavs Hospital, Trondheim University Hospital, Trondheim, Norway

**Keywords:** Pregnant, Physical activity, Maternal risks, Diabetes, High BMI

## Abstract

**Background:**

The primary aim was to investigate if supervised exercise training during pregnancy could reduce postpartum weight retention (PPWR) three months after delivery in overweight and obese women. We also measured circulating markers of cardiometabolic health, body composition, blood pressure, and physical activity level.

**Methods:**

This was a secondary analysis of a randomised controlled trial in which 91 women with BMI ≥ 28 kg/m^2 ^were allocated 1:1 to an exercise program or a control group. Women in the exercise group were prescribed three weekly, supervised sessions of 35 min of moderate intensity walking/running followed by 25 min of resistance training. The control group received standard maternal care. Assessments were undertaken in early pregnancy, late pregnancy, and three months postpartum. PPWR was defined as postpartum body weight minus early pregnancy weight.

**Results:**

Seventy women participated three months after delivery, and PPWR was −0.8 kg in the exercise group (*n* = 36) and −1.6 in the control group (*n* = 34) (95% CI, −1.83, 3.84, *p* = 0.54). Women in the exercise group had significantly lower circulating insulin concentration; 106.3 pmol/l compared to the control group; 141.4 pmol/l (95% CI, −62.78, −7.15, *p* = 0.01), and showed a tendency towards lower homeostatic measurement of insulin resistance (HOMA2-IR) (3.5 vs. 5.0, 95% CI, −2.89, 0.01, *p* = 0.05). No women in the exercise group compared to three women in the control group were diagnosed with type 2 diabetes postpartum (*p* = 0.19). Of the women in the exercise group, 46.4% reported of exercising regularly, compared to 25.0% in the control group (*p* = 0.16).

**Conclusions:**

Offering supervised exercise training during pregnancy among overweight/obese women did not affect PPWR three months after delivery, but reduced circulating insulin levels. This was probably due to a higher proportion of women being active postpartum in the exercise group.

**Trial registration:**

ClinicalTrials.gov (NCT01243554), registration date: September 6, 2010.

**Electronic supplementary material:**

The online version of this article (10.1186/s12884-017-1653-5) contains supplementary material, which is available to authorized users.

## Background

Overweight and obesity among women in fertile age are associated with adverse health outcomes for mother and child, both during pregnancy and postpartum [1–11]. Overweight is defined as body mass index (BMI) ≥ 25 kg/m^2^, and obesity as BMI ≥ 30 kg/m^2^ (according to the WHO classification system) [12]. Pre-pregnancy overweight and obese women are at increased risk for high postpartum weight retention (PPWR) [10, 11]. Obese women are two times more likely than normal weight women to exceed the Institute of Medicine’s (IOM) recommendations for gestational weight gain [13], and half of the PPWR can be explained by excessive gestational weight gain [14]. High PPWR is associated with reduced insulin sensitivity, hypertension, and later development of type 2 diabetes mellitus and cardiovascular disorders [7, 8, 15, 16]. High PPWR also predisposes for high pre-pregnancy BMI [7, 8] and further reduced maternal metabolic function in future pregnancies [17].

Finding lifestyle interventions to limit gestational weight gain and PPWR among overweight and obese women is important. Previous research investigating the effect of lifestyle programs during pregnancy targeting this group of women have demonstrated conflicting results on PPWR, glucose tolerance, and other cardiometabolic health variables [18–21]. Further, few trials have investigated the effect of regular exercise during pregnancy as the only intervention.

The primary aim of the Exercise Training in Pregnancy (ETIP) trial was to assess if offering supervised exercise training during pregnancy would reduce gestational weight gain in women with pre-pregnancy BMI of ≥28.0 kg/m^2^ [22, 23]. At delivery we found no difference in gestational weight gain between the groups, but we observed a lower incidence of gestational diabetes mellitus (GDM) and lower blood pressure in the exercise group [22].

This is a secondary analysis of data from the ETIP trial where we assessed if providing a supervised exercise program during pregnancy could reduce PPWR three months after delivery. Our a priori hypothesis was that the women in the exercise group would have a lower PPWR. We also investigated effects of the intervention during pregnancy on body composition, blood pressure, physical activity level, and various circulating markers of cardiometabolic health three months postpartum.

## Methods

### Trial design

The ETIP trial was a single-centre, parallel-group randomised controlled trial (RCT) investigating effects of offering supervised regular exercise training during pregnancy compared to standard maternal care only, in overweight and obese women. The primary outcome measure in ETIP was gestational weight gain. The trial was conducted between September 2010 and March 2015 at the Norwegian University of Science and Technology (NTNU) and St. Olavs Hospital, Trondheim University Hospital, Norway. The study was approved by the Regional Committee for Medical and Health Research Ethics (REK-midt 2010/1522), registered in ClinicalTrials.gov (NCT01243554) and was in accordance with the Helsinki Declaration of 1975. The ETIP study protocol and primary findings of the trial have been published previously [22, 23].

We experienced slow recruitment in the trial and made changes to the study protocol after commencement of the trial to accommodate the need for more participants [22]. We experienced eligible women making contact for participation too late for randomisation within gestational week 16, therefore the originally criterion for maximum inclusion time in gestational week 16 was changed to gestational week 18 in November 2012. The inclusion criterion pre-pregnancy BMI was changed from ≥ 30 kg/m^2^ to ≥ 28 kg/m^2^ in March 2013, in attempt to increase the number of women eligible for participation [22]. The changes were approved by the Regional Committee for Medical and Health Research Ethics.

### Participants

Women were eligible if they had a pre-pregnancy BMI ≥ 28, age ≥ 18 years, gestational week < 18, carrying one singleton live foetus at the 11–14-week ultrasound scan, and were able to attend assessments and exercise sessions at St. Olavs Hospital. Exclusion criteria were; habitual exercise training (twice or more weekly) in the period before pregnancy, high risk for preterm delivery, diseases that could interfere with participation, and contraindications in accordance to The American College of Obstetricians and Gynecologists (ACOG) recommendations for physical activity and exercise during pregnancy [24, 25]. The women received written information and signed informed consent on behalf of themselves and their foetus before participation and randomisation.

### Intervention

All participants received standard maternal care. In addition, women in the exercise group were offered supervised exercise sessions three times per week at the hospital from time of inclusion (at gestational week 12–18) until delivery [22]. The exercise program provided was in accordance with the recommendations from ACOG [26]. Women in the exercise group walked or ran on treadmills for 35 min at moderate intensity (65–80% of maximal capacity, estimated using a rate of perceived exertion of 12–15 on the Borg 6–20 scale [27]), followed by 25 min of resistance exercises for large muscle groups and a strength training program for the pelvic floor muscles. The strength training consisted of weight-bearing exercises such as squats, push-ups, diagonal lifts on all fours, oblique abdominal crunches, and pelvic floor muscle exercises, with three sets of ten repetitions of each exercise separated by one minute rest between sets. In addition, the women also performed three sets of the “plank exercise” for 30 s. A physical therapist supervised all sessions and registered each woman’s adherence to the program. We also advised women in the exercise group to do 35 min of endurance exercise and 15 min of resistance exercise at home at least once weekly, as well as daily pelvic floor muscle strengthening exercises. The participants’ exercise sessions at the hospital, including duration, intensity and possible adjustments, were registered by study personnel. The participants’ registered their home-based exercise and general physical activities in a training diary. The women in the exercise group were informed of recommended weight gain during pregnancy, based on the guidelines of the Institute of Medicine (IOM) [28]. They received an individually adjusted weight gain curve, where they weekly registered their weight measured at the hospital. The supervised exercise sessions were terminated at delivery. Women in the control group were informed about the recommended level of physical activity during pregnancy and were not discouraged from exercising on their own.

### Outcomes

The principal outcome of this secondary analysis was PPWR, defined as body weight (kg) at the postpartum visit minus body weight (kg) at early pregnancy. Weight was measured using a calibrated electronic scale (SECA 770, Medema, Norway). We also assessed the difference between postpartum weight and self-reported weight before pregnancy.

All participants underwent the same test protocol at early pregnancy (gestational week 12–18), in late pregnancy (gestational week 34–37), and three months postpartum. The participants fasted overnight for ≥ 10 h before undergoing an oral glucose tolerance test where they drank 75 g of glucose dissolved in 2.5 dl of water. We report the number of women who fulfilled the WHO definition of type 2 diabetes; fasting plasma glucose ≥ 7.0 mmol/l, and/or 2 h concentration ≥ 11.1 mmol/l. [29] We measured plasma insulin by enzyme immunoassay (ELISA, IBL-International, Germany), using a DS2 ELISA processing system (Dynex Technologies, USA), according to the manufacturer’s procedures. As a measure of insulin resistance, we used the homeostatic assessment of insulin resistance (HOMA2-IR), calculated as [glucose*insulin]/22.5 [30]. All other blood measurements were analysed by Roche Modular P-system (Roche, Switzerland).

Blood pressure measurements were taken three times at two-minute intervals, and the average was used in the analysis. Hypertension was defined as a systolic blood pressure ≥ 140 mmHg and/or a diastolic blood pressure ≥ 90 mmHg. We used a Harpenden Caliper (Holtain Ltd., UK) to measure subscapular-, biceps-, and triceps skinfold thickness. Body composition was additionally measured with air displacement plethysmography (BOD POD, COSMED The Metabolic Company, Italy). We measured waist circumference at the postpartum visit, using measuring tape at the level of the umbilicus at normal expiration. Assessments were undertaken by principal investigators (KKG and TM), trained nurses and biomechanical laboratory personnel. For a more detailed description of outcome measures, see Garnæs et al. [22]

At the three months postpartum visit, as well as in early and late pregnancy, the participants answered questionnaires about their physical activity and exercise training. They were asked if they adhered to the recommendations of ≥ 150 min of moderate intensity physical activity per week, and about their amount and intensity of exercise training. Women were also asked about breastfeeding at the time of the postpartum visit; whether they were exclusively breastfeeding and the number of meals per 24 h.

### Sample size

The sample size calculation in the ETIP-trial was based on a primary outcome of gestational weight gain from baseline to delivery [22, 23]. To detect a 6 kg clinically significant difference in weight gain between the groups, we needed a minimum of 118 participants (with alpha 0.05, beta 0.90). We did not do a separate power calculation for the analyses presented in this report.

### Randomisation and blinding

After early pregnancy assessments were undertaken, we allocated participants 1:1 to the exercise or the control group, using a computer random number generator. The participants allocated to the intervention were invited to exercises sessions immediately after randomisation. For details about the randomisation procedure, see Garnæs et al. [22] The personnel measuring weight at birth and undertaking blood analyses and the statistician were blinded for group allocation. All other measurements were unmasked. To limit bias were blinding was not possible, detailed test-protocols were used, and a low number of- and the same study personnel performed the assessments in the trial.

### Statistical methods

Analyses were done according to the “intention to treat” principle. All available data was used at all time points. Baseline data (early pregnancy) was tested for normality and compared between groups by independent samples t-tests and Fisher’s Exact Tests. The effect of treatment on the continuous postpartum outcomes was assessed with mixed linear models. The effect of time and treatment was specified as a fixed effect having the levels ‘baseline’, ‘training late pregnancy’, ‘control late pregnancy’, ‘training postpartum’ and ‘control postpartum’. No systematic differences between groups at baseline were assumed due to randomisation. Participant ID was included as a random effect to account for repeated measurements. To account for apparent variance heterogeneity across time, the covariance structure for the error term was specified as diagonal. The effect of treatment on dichotomous postpartum outcomes was analysed using exact logistic regression adjusted for the baseline (early pregnancy) outcome when available, with the exercise group as the reference group.

Analyses were performed using IBM SPSS Statistics 22 for baseline values, R version 2.13.1 for continuous outcome data, and Stata version 13.1 for dichotomous outcome data. All results are given as mean values with 95% confidence intervals and *p*-values < 0.05 were considered as significant.

We did supplementary mixed model analyses of PPWR where we adjusted for the number of days since delivery, lactation and physical activity. We also investigated association between PPWR and gestational weight gain, lactation and physical activity. We performed, as described in the protocol [23], per protocol analyses of women in the exercise group adhering to the exercise protocol. In these analyses we included women in the exercise group who undertook one of the following: 1) attending ≥ 42 organized exercise sessions, 2) attending ≥ 28 exercise sessions + performing ≥ 28 home exercise sessions, 3) performing ≥ 60 home exercise sessions. The exercise had to be ≥ 50 min of aerobic and/or strength training to count as a home session.

## Results

**Fig. 1:**
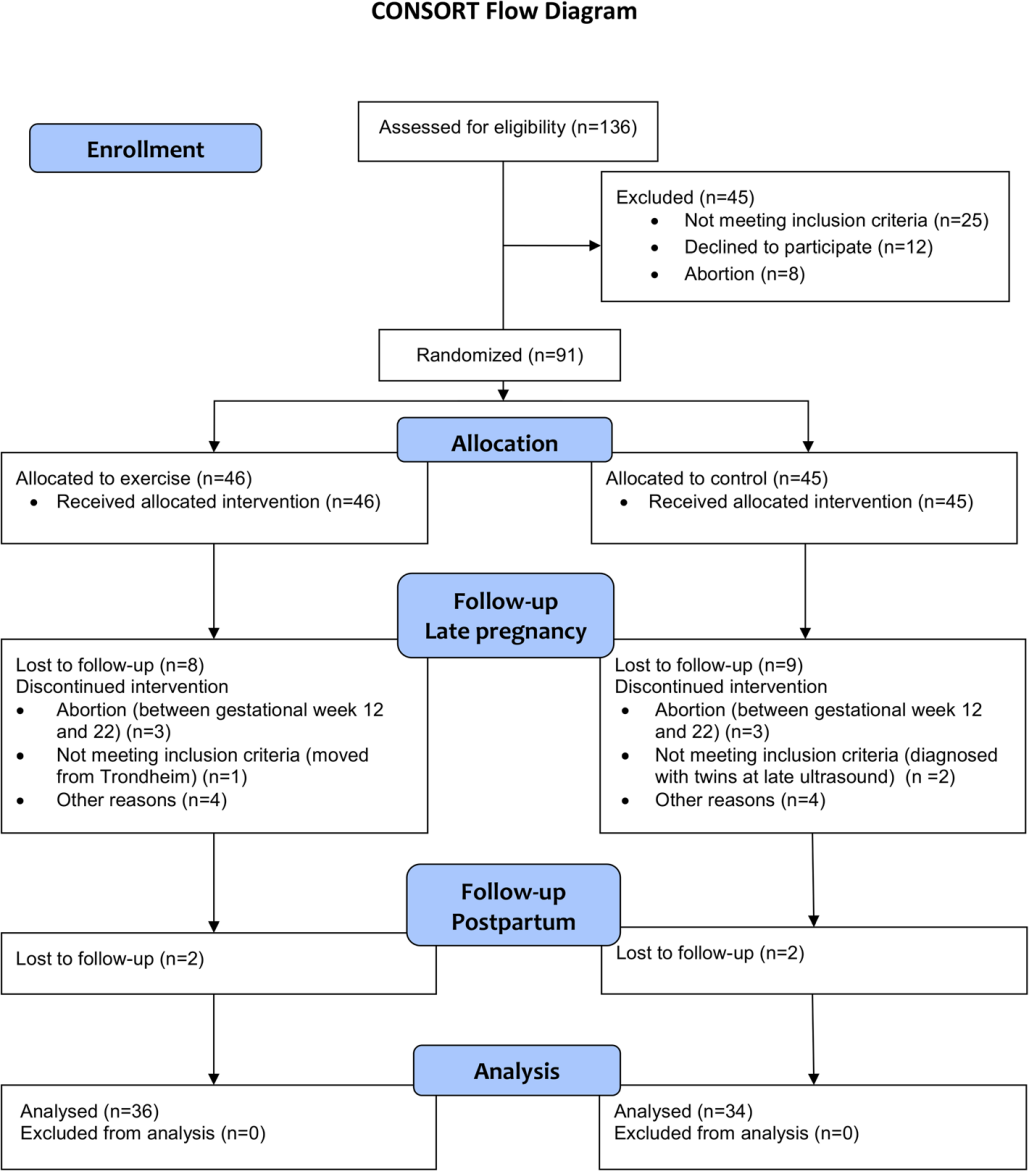
Flow chart of the ETIP trial

The trial was conducted between September 2010 and March 2015, and enrolment was ended due to prolonged time for inclusion and fewer eligible participants than expected. Figure 1 outlines the flow of participants during the trial. Seventy (77%) of 91 women included in the ETIP trial were assessed at three months postpartum and two women in each group dropped out from late pregnancy/delivery until the postpartum visit (Figure 1). Table 1 shows participants’ demographic characteristics at baseline. Additional baseline characteristics of the whole sample in the ETIP trial has been published previously [22]. Apart from a lower fasting glucose in the exercise group (*p =* 0.02), baseline (early pregnancy) characteristics did not differ between groups. Of the women in the exercise group included in the postpartum analyses, 54.3% adhered to the training protocol. The mean time for postpartum testing was 99.8 ± 10.2 days after delivery in the exercise group and 95.7 ± 10.4 days in the control group. The women in the exercise group performed 31.7 ± 15.3 (range 0–53) supervised sessions at the hospital, and 19.2 ± 16.5 (range 0–72) exercise sessions at home. Mean gestational weight gain during pregnancy was 10.5 ± 4.6 kg in the exercise group and 9.7 ± 6.9 kg in the control group (*p* = 0.55). Among women in the exercise group, 58% gained more weight during pregnancy than recommended by the IOM guidelines compared with 44% in the control group [22]. Table 2 presents model-based outcomes at baseline (means for all participants) and at the postpartum visit.

**Table 1:** Participants’ demographic characteristics at baseline (early pregnancy). Observed data presented as mean ± standard deviation or number of participants (percent)

Participant characteristics at baseline	Exercise Group (*n =* 36)	Control Group (*n =* 34)	
	Mean ± SD	Mean ± SD	*p*-value
Age (years)	31.6 ± 3.6	31.3 ± 4.6	0.73
Weight (kg)	94.7 ± 12.4	99.3 ± 14.4	0.16
Height (cm)	167.0 ± 5.7	167.8 ± 6.3	0.59
BMI (kg/m^2^)	33.9 ± 3.8	35.2 ± 4.5	0.20
	n (%)	n (%)	
Weight classification			0.35
Overweight, BMI 28.0–29.9 kg/m^2^	2 (5.6%)	3 (8.8%)	
Class 1 obesity, BMI 30.0–34.9 kg/m^2^	23 (63.9%)	15 (44.1%)	
Class 2 obesity, BMI 35.0–39.9 kg/m^2^	9 (25.0%)	11 (32.4%)	
Class 3 obesity, BMI ≥ 40.0 kg/m^2^	2 (5.6%)	5 (14.7%)	
Parity			0.71
0	18 (50.0%)	15 (44.1%)	
1	15 (41.7%)	14 (41.2%)	
2	3 (8.3%)	4 (11.8%)	
≥ 3	0 (0.0%)	1 (2.9%)	
Current smoking	2 (5.6%)	4 (11.8%)	
Education			0.85
Primary/secondary school	0 (0.0%)	0 (0.0%)	
High school	9 (25.0%)	6 (18.2%)	
University ≤ 4 y	13 (36.1%)	10 (29.4%)	
University > 4 y	14 (38.9%)	17 (50.0%)	
Currently employed	32 (88.9%)	26 (76.5%)	0.21

Missing: Education: Control group: 1

Statistics: Current Smoking and Currently employed were analysed by Fisher’s Exact Test. Weight classification, Parity and Education were analysed by Pearson Chi-Square Test

*Abbreviations*: *BMI* Body Mass Index

**Table 2:** Outcomes at three months postpartum. “Intention to treat” model based analyses with early pregnancy (baseline) mean for all participants, and comparisons between groups at postpartum presented as mean, 95% confidence interval (CI) and p-value. Weight retention was estimated based both on the difference between postpartum weight and early pregnancy (baseline) weight, and between postpartum weight and self-reported pre-pregnancy weight

Outcomes at postpartum	Baseline	Exercise Group (*n =* 36)		Control Group (*n =* 34)		Between-Group Comparison		
		Mean	95% CI	Mean	95% CI	Mean Diff	95% CI	*p*-value
Weight (kg)	96.8	96.0	92.7, 99.3	95.2	91.9, 98.5	0.82	−1.83, 3.46	0.54
PPWR^1^ (kg)^a^		- 0.8	−2.7, 1.1	−1.6	−3.5, 0.3	0.82	−1.83, 3.84	0.54
PPWR^2^ (kg)^b^		1.52	−0.73, 3.78	0.52	−1.82, 2.86	1.0	−2.15, 4.16	0.53
BMI (kg/m^2^)	34.5	34.2	33.2, 35.3	33.9	32.9, 35.0	0.29	−0.67, 1.25	0.55
Waist circumference (cm)	107.5	105.0	101.7, 108.2	102.9	99.6, 106.2	2.06	−1.34, 5.47	0.24
Body composition^c^								
Fat mass (kg)	43.1	42.1	39.6, 44.6	42.0	39.5, 44.5	0.11	−2.05, 0.92	0.92
Fat mass (%)	44.6	44.2	43.0, 45.5	43.9	42.7, 45.2	0.28	−0.83, 1.38	0.62
Fat-free mass (kg)	52.7	52.1	50.4, 53.7	53.0	51.4, 54.7	−0.96	−2.83, 0.92	0.32
Fat-free mass (%)	55.4	55.7	54.4, 57.1	56.4	55.1, 57.7	−0.65	−1.97, 0.67	0.33
Skinfold thickness								
Biceps area (mm)	21.1	16.7	14.7, 18.7	17.5	15.5, 19.6	−0.85	−3.15, 1.44	0.47
Triceps area (mm)	30.0	26.4	24.4, 28.4	26.8	24.8, 28.8	−0.43	−2.67, 1.82	0.71
Subscapular area (mm)	31.8	28.5	26.3, 30.8	30.0	27.7–32.3	−1.44	−4.03, 1.16	0.28
Blood pressure								
Systolic BP (mm/Hg)	124.5	120.6	117.5, 123.8	124.02	120.7, 127.4	−3.40	−7.70, −0.99	0.13
Diastolic BP (mm/Hg)	76.0	75.8	73.3, 78.4	78.4	75.7, 81.1	−2.61	−6.19, 0.96	0.15
Blood measurements								
Fasting glucose (mmol/l)	4.7	5.1	4.9, 5.3	5.1	4.8, 5.26	0.02	−0.24, 0.27	0.91
120-min glucose (mmol/l)	5.9	5.2	4.7, 5.8	5.8	5.3, 6.4	−0.60	−0.60, 1.31	0.10
Insulin (pmol/l)	139.6	106.3	83.3, 129.2	141.4	118.1, 164.6	−35.10	−62.78, −7.15	*0.01*
HbA1c (%)	5.2	5.3	5.2, 5.4	5.4	5.3, 5.5	−0.10	−0.25, 0.04	0.17
Insulin C-peptide (nmol/l)	0.6	0.7	0.6, 0.8	0.7	0.6, 0.8	−0.01	−0.14, 0.11	0.82
Triglycerides (mmol/l)	1.4	0.8	0.6, 1.0	1.0	0.8, 1.2	−0.21	−0.47, 0.05	0.12
Ferritin (pmol/l)	127.0	69.9	56.0, 83.6	64.5	49.7, 79.1	5.46	−14.81, 25.71	0.60
HDL cholesterol (mmol/l)	1.7	1.5	1.5, 1.6	1.5	1.4, 1.6	−0.02	−0.14, 0.11	0.81
LDL cholesterol (mmol/l)	2.8	3.1	2.8, 3.4	3.2	2.8, 3.5	−0.08	−0.51, 0.34	0.71
Total cholesterol (mmol/l)	5.0	4.9	4.5, 5.2	5.1	4.7, 5.4	−0.23	−0.71, 0.25	0.34
Haemoglobin (g/l)	126.7	128.4	125.4, 131.5	129.7	126.5, 132.9	−1.30	−5.50, 2.90	0.56
High-sensitive CRP (mg/l)	10.7	4.2	2.9, 5.6	4.8	3.4, 6.2	−0.58	−2.50, 1.34	0.55
HOMA2-IR	2.5	3.5	2.5, 4.6	5.0	3.9, 6.0	−1.44	−2.89, 0.01	0.05

Missing: The number of missing in the exercise group varied between 1and 5, in the control group between 1 and 3

Statistics: The effect of treatment was assessed with linear mixed models. For the primary and secondary outcomes, the effect of time and treatment was taken as a fixed effect. Due to randomisation, no systematic differences between groups at baseline were assumed. Insulin was significantly lower (*p* = 0.01) in th exercise group postpartum

*Abbreviations*: *PPWR* postpartum weight retention, *BMI* Body mass index, *BP* blood pressure, *HbA1c* Glycated Haemoglobin, *HDL* High-density lipoprotein, *LDL* Low-density lipoprotein, *CRP* C-reactive protein, *HOMA2-IR* homeostatic assessment of insulin resistance

^a^PPWR^1^, postpartum weight minus weight at early pregnancy (baseline)

^b^PPWR^2^, postpartum weight minus pre-pregnancy weight. Weight at pre-pregnancy was based on self-reported data. Mean pre-pregnancy weight for all participants were 94.4 kg

^c^Body composition was measured by air displacement plethysmography (BOD POD)

### Postpartum weight retention

PPWR was not significantly different between groups, with −0.8 kg in the exercise group and −1.6 kg in the control group (*p* = 0.54) (Table 2). Women in both groups had returned to their early pregnancy body weight three months postpartum. We observed no association between PPWR and gestational weight gain (*p* = 0.79), PPWR and lactation (*p* = 0.63), or between PPWR and fulfilling recommendations of 30 min of physical activity per day (*p* = 0.20).

### Other outcome measures

**Fig. 2:**
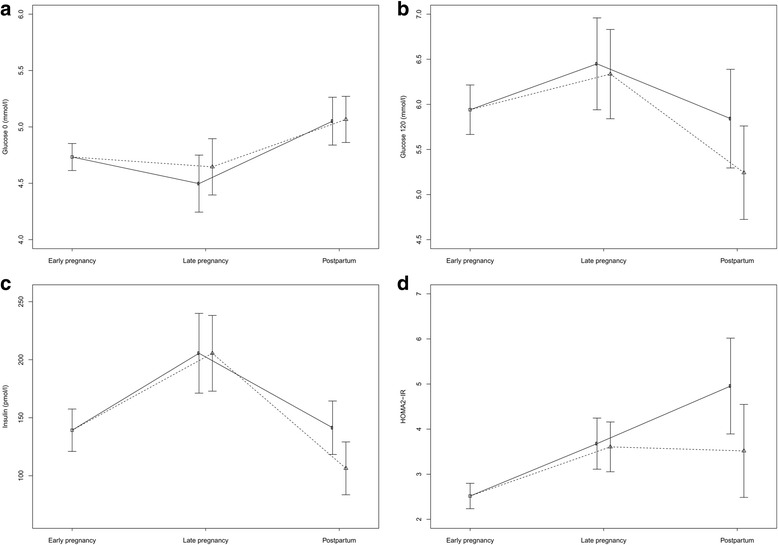
**a** Fasting glucose at early pregnancy, late pregnancy, and postpartum. **b** 120 min glucose after an oral glucose tolerance test at early pregnancy, late pregnancy, and postpartum. **c** insulin at early pregnancy, late pregnancy, and postpartum. **d** homeostatic measurement of insulin resistance (HOMA2-IR) at early pregnancy, late pregnancy, and postpartum

Fasting glucose was equal between groups at the postpartum visit (Fig. 2a), but we observed a tendency towards lower 120 min glucose in the exercise group compared to the control group (5.2 mmol/l vs 5.8 mmol/l, *p* = 0.10) (Fig. 2b). The insulin concentration was significantly lower in the exercise group compared to the control group (*p* = 0.01) (Table 1). Figure 2c outlines the insulin levels at baseline, in late pregnancy, and postpartum. HOMA2-IR (insulin resistance) was lower in the exercise group, but the group difference was not statistically significant (Fig. 2d). No women in the exercise group compared to three women in the control group fulfilled the diagnostic criteria for type 2 diabetes postpartum (*p* = 0.19, Table 2). All three women were diagnosed with GDM in late pregnancy, and none had diabetes before pregnancy. We also observed a trend towards lower systolic and diastolic blood pressure in the exercise group (Table 1). Approximately 75% of the total study population fulfilled the recommended amount of weekly general physical activity three months postpartum (Table 3). Twice as many women in the exercise group reported regular exercise (defined as ≥ 90 min with moderate intensity and/or ≥ 45 min with high intensity per week) postpartum, but the between-group difference was not statistically significant. The number of women exclusively breastfeeding at the postpartum visit was not significantly different between groups (Table 3).

**Table 3:** Outcomes at three months postpartum. “Intention to treat” analysis, observed data, for the exercise and the control group and comparison between groups are presented in number of participants (N), percentage (%), odds ratio (OR), 95% confidence interval (CI), and p-value

Outcomes at postpartum	Exercise group *n* = 36	Control group *n* = 34	Between-Group Comparison		
	n (%)	n (%)	Odds Ratio	95% CI	*p*-value
Type 2 diabetes	0 (0)	3 (9.1)	4.96	0.46, ∞	0.19
Hypertension	3 (8.8)	3 (10.0)	1.17	0.15, 9.30	1.00
Physical activity ≥ 150 min/week^a^	21 (72.4)	22 (78.6)	1.17	0.68, 2.02	0.76
Exercise training^b^	13 (46.4)	7 (25.0)	0.39	0.12,1.19	0.16
Exclusively breastfeeding	18 (60.0)	21 (77.8)	1.44	0.90, 2.31	0.17
Breastfeeding 3–4 meals/24 h	4 (13.3)	1 (3.7)	0.63	0.37, 1.05	0.36

Missing: Type 2 diabetes: Exercise group 1 missing, control group 1 missing. Hypertension: Exercise group 1 missing, control group 4 missing. Physical activity questionnaire: Exercise group 7 missing, control group 6 missing. Lactating questionnaire: Exercise group 6 missing, control group 7 missing

Statistics: Type 2 diabetes and hypertension were analysed by exact logistic regression Model. Data on physical activity and breastfeeding are based on a self-reported questionnaire and were analysed by Fisher’s Exact test

Definitions: Type 2 diabetes: Fasting plasma glucose ≥7.0 mmol/l or 2 h concentration ≥ 11.1 mmol/l, according to the definition of the World Health Organization (WHO). Hypertension: Systolic blood pressure ≥ 140, diastolic blood pressure ≥ 90

^a^Physical activity ≥150 min/week: 30 min of daily physical activity

^b^Exercise training ≥90 min with moderate intensity and/or ≥45 min with high intensity per week

### Additional analyses

We analysed PPWR in both groups adjusted for number of days from birth to postpartum test and observed no significant effect on postpartum weight (*p* = 0.76) or PPWR (*p* = 0.32). The effect estimate of weight loss per day was −0.016 kg. We found no effect of adjusting for lactation and physical activity. Half of the exercising women included in the postpartum analysis (*n* = 19) fulfilled the training intervention during pregnancy as described in the study protocol [23]. Detailed data are presented in Additional file 1 and Additional file 2. We found no difference in PPWR (postpartum minus early pregnancy) between the per protocol exercise group (−0.1 kg, 95% CI, −2.7, 1.1) and the control group (−1.7 kg, 95% CI, −3.5, 0.3) (*p* = 0.35), and no difference in PPWR when using self-reported pre-pregnancy weight between the per protocol exercise group (2.5 kg, 95% CI, −0.7, 3.8) and the control group (0.2 kg, 95% CI, −1.8, 2.9) (*p* = 0.28). At postpartum, there was no difference in mean weight between the exercise group (95.9 kg) and the control group (94.8 kg) (*p* = 0.47) (Additional file 1). Women in the per protocol exercise group had significantly lower resting systolic and diastolic blood pressure compared to the control group, (117.0/73.1 mmHg vs. 124.0/78.4 mmHg) (systolic BP, *p* = 0.01, diastolic BP, *p* < 0.01), and they had lower insulin levels (14.1 mmol/l vs 19.7 mmol/l, *p* = 0.04) (Additional file 1). No harmful, unintended or adverse events were reported.

## Discussion

Offering supervised regular exercise during pregnancy for overweight and obese women did not lower PPWR compared to women receiving standard maternal care. Both groups regained the pre-pregnancy weight three months after delivery. However, we found a significantly lower blood insulin concentration and a tendency towards lower HOMA2-IR in the exercise group compared to the control group. These findings may indicate a reduced risk for developing type 2 diabetes among the exercising women, however, studies with higher power is needed to confirm this. Among women who adhered to the training protocol during pregnancy, we also found significantly lower systolic and diastolic blood pressure three months postpartum.

Pre-pregnancy BMI is a strong predictor of PPWR with higher weight retention in overweight and obese women [31, 32]. We have found no RCTs assessing the isolated effects of exercise training in pregnancy on PPWR in exclusively overweight and obese women. Previous studies have combined different types of intervention, such as diet and exercise, and/or included participants of all BMI categories. Those RCTs have shown divergent results; some have found no effect [33–36], whereas others have found lower PPWR in the intervention group [32, 37]. Phelan and colleagues [32], found lower PPWR in normal weight and overweight women after a lifestyle intervention program, but not among obese women. According to a meta-analysis by  Nascimento and colleagues, trials reporting positive effects on PPWR are characterised by including women in all BMI categories and have combined supervised exercise training and intensive dietary interventions [21]. Among studies providing ancillary analyses, some have suggested positive effects of exercise on PPWR among women adhering to the intervention protocol [32–35]. We did not show any differences in PPWR between groups using early pregnancy weight measurement, self-reported weight pre-pregnancy, or analysing women who exercised per protocol. However, women in both groups had almost regained their pre-pregnancy and early pregnancy weight at the postpartum visit. Our trial included supervised exercise training from early pregnancy and throughout the pregnancy, but the results indicate that the amount or intensity of the exercise ought to be higher or combined with dietary intervention to improve outcomes. Adherence to the training protocol was low, and may have reduced the difference between groups. Low adherence to the training protocol is a common challenge in trials including obese pregnant women.

Obese women have an increased risk for high insulin values and for developing diabetes mellitus type 2 postpartum [38]. We found significantly lower concentration of insulin in the exercise group compared to the control group. During pregnancy the insulin resistance increases, especially in obese women [39, 40]. To compensate, increased insulin secretion is needed [39]. Lower insulin, and trends towards lower 120 min glucose level and lower HOMA2-IR among women in the exercise group, may indicate lower risk of developing type 2 diabetes. The difference between groups in incidence of type 2 diabetes was not significant, but our results may have been affected by a small sample size.

Obese women are at increased risk for high blood pressure during pregnancy and postpartum [41–43]. We observed significantly lower systolic and diastolic blood pressure among women who adhered to the prescribed exercise, compared to the control group. We are not aware of any previous trial assessing the effect of exercise training during pregnancy on postpartum blood pressure. However, exercise has been shown to lower resting blood pressure among obese, non-pregnant subjects [44, 45].

Women are recommended to be physically active during pregnancy and postpartum to maintain a healthy weight and to prevent negative health outcomes [12, 46]. However, physical activity tends to decrease significantly during these periods, especially among women with BMI ≥ 25 kg/m^2^ [47]. About 60% of the women in the ETIP trial (in both groups) reported to be physically active, whereas 77% in the exercise group versus 23% in the control group, reported to exercise regularly in late pregnancy [22]. At the postpartum visit, approximately 75% of all women in our study reported fulfilling the recommendations of minimum 150 min of weekly moderate intensity physical activity. A higher proportion of women in the exercise group (46% vs 25%) reported regular exercise postpartum. These results show that the amount of general physical activity is equal between groups both during the pregnancy and after delivery, but with a tendency of more structured exercise training in the exercise group also postpartum. Concerning the inclusion criteria “not exercising regularly pre-pregnancy”, these numbers show an increase in exercise training for both groups postpartum compared to before pregnancy. A low number of participants in each group likely hampers the statistical comparison between groups.

Gestational weight gain and lactation are important factors for PPWR [32, 48]. In the present study, we found no associations between gestational weight gain and PPWR or between lactation and PPWR.

### Study strength and limitations

The ETIP trial had a randomised, controlled study design. The exercise program was described in detail and should be easy to reproduce. Previous research has found supervised exercise to be important for adherence to the exercise protocol, for motivation, and for the safety of the participant, and to be more effective than general guidance [18]. We measured weight objectively at study entry and at postpartum, and we only included sedentary overweight/obese women in the trial. We measured skinfold thickness and body composition in addition to weight, and provided information on potential confounding factors such as lactation and GWG. Our intervention included the exercise program only, and no diet. Thus, we could assess the effect of exercise alone in contrast to previous trials with mixed interventions.

The main limitation was a small study sample. We did not recruit as many participants as originally planned and experienced additional drop-outs during the intervention period. This affected the power of the study and decreased the possibility of detecting true effects of the intervention. The proportion of drop-outs was, however, equally distributed between groups, and we had only two drop-outs in each group after delivery. In addition, only 50% of women in the exercise program adhered to the protocol. We report exercise intensity only by the rate of perceived exertion in this trial, due to the lack of a precise estimate of maximum heart rate for each participant. Since pregnancy can influence the heart rate during exercise [49], we chose not to include data on heart rate during exercise. We prolonged the time limit for inclusion in the trial from gestational week 16 to 18, which reduced the mean number of weeks of exercise before delivery, and thus the effect of the intervention. Our change in inclusion criteria BMI from ≥ 30 to ≥ 28 kg/m^2^, may have reduced the homogeneity of the trial population, but only five women in the postpartum analysis had a pre-pregnancy BMI below 30 kg/m^2^.

The current trial did not provide any information on diet and possible changes in eating habits in the groups. The control group underwent comprehensive health assessments during pregnancy and after delivery and this may have motivated also the women in the control group to undertake lifestyle changes.

### Generalisability

The participants were recruited from Google advertisement and through an information letter to all pregnant women in Trondheim. There is a risk for over-representation of highly motivated women in the trial. This may influence the external validity (generalizability) of the trial, but not the internal validity (comparisons between groups).

### Clinical relevance

The exercise intervention in the current trial was based on the ACOG recommendations for physical activity and exercise during pregnancy. The program required no equipment and consisted of exercises that could easily be implemented by women themselves at home. The findings are relevant for sedentary overweight and obese pregnant women. The finding of lower concentration of insulin in the exercise group is clinically important as this may reduce the risk of future type 2 diabetes [50]. The blood pressure was lower in the women who reported to exercise per protocol during pregnancy and imply a reduced risk for developing cardiovascular diseases. This highlights the need of increasing adherence to exercise training in pregnancy for this population. No adverse events related to exercise occurred, and the findings in the current trial support the recommendations for exercise training during pregnancy.

## Conclusion

Offering supervised exercise during pregnancy among overweight and obese women did not affect PPWR three months after delivery compared to standard antenatal care. Both groups had regained their early pregnancy weight three months postpartum. We observed lower circulating insulin among the women in the exercise group, as well as lower blood pressure in those who adhered to the exercise protocol. These findings may decrease the risk for developing both type 2 diabetes and cardiovascular diseases later in life. Further studies are needed to assess if supervised exercise during pregnancy can reduce the risk for development of type 2 diabetes and hypertension postpartum.

## Additional files

Additional file 1: Table S1. Per protocol analysis of contiruous outcome variables at three months postpartum. (PDF 191 kb)

Additional file 2: Table S2. Per protocol analysis of categorical outcome vairables at three months postpartum. (PDF 161 kb)

## Abbreviations

ACOG: American College of Obstetricians and Gynecologists
BMI: Body mass index (kg/m^2^)
ETIP: Exercise training in pregnancy
GDM: Gestational diabetes mellitus
IOM: Institute of Medicine
OGTT: Oral glucose tolerance test
PPWR: Postpartum weight retention
RCT: Randomised controlled trial
WHO: World Health Organization
