# Supplementary material for: Exercise training during pregnancy reduces circulating insulin levels in overweight/obese women postpartum: secondary analysis of a randomised controlled trial (the ETIP trial)
Source: BMC Pregnancy Childbirth. 2018 Jan 8;18:18. doi: 10.1186/s12884-017-1653-5 (PMC5759335; doi:10.1186/s12884-017-1653-5)
Supplement: Supplementary file 1 — Per protocol analysis of contiruous outcome variables at three months postpartum. (PDF 191 kb) [file 12884_2017_1653_MOESM1_ESM.pdf]

Additional Table 1. Outcomes at three months postpartum. "Per protocol" model based analyses with baseline mean (all participants at early pregnancy) and comparison between groups are presented as mean, 95% confidence interval (CI) and p-value. Weight retention is estimated based both on the difference between postpartum weight and early pregnancy weight, and between postpartum weight and pre-pregnancy weight.

		**Per protocol Exercise Group (*n* = 19)**		**Control Group (*n* = 34)**		**Between-Group Comparison**		
	**Baseline Mean**	**Final Mean**	**95% CI**	**Final Mean**	**95% CI**	**Mean Diff**	**95% CI**	***p*-Value**
**Weight (kg)**	96.3	95.9	91.8, 100.1	94.8	91.0, 98.5	1.18	-2.05, 4.42	0.47
**PPWR[1] (kg)***		- 0.1	-3.0, 2.2	-1.7	-3.5, 0.4	1.64	-1.82, 5.11	0.35
**PPWR[2] (kg)****		2.5	-1.5, 4.8	0.2	-1.8, 3.1	2.32	-1.98, 6.61	0.28
**BMI (kg/m^2)**	34.6	34.5	33.1, 35.8	34.0	32.8, 35.2	0.48	-0.70, 1.66	0.42
**Waist circumference (cm)**	106.5	99.3	95.6, 103.1	104.4	99.9, 109.0	-5.10	-11.66, 1.46	0.13
**Body composition*****								
Fat mass (kg)	43.1	42.3	39.1, 44.9	42.0	39.2, 44.9	0.28	-2.46, 3.01	0.84
Fat mass (%)	44.8	44.3	42.7, 45.9	44.08	42.7, 45.5	0.22	-1.15, 1.59	0.76
Fat-free mass (kg)	52.3	51.9	49.8, 54.0	52.6	50.9, 54.4	-0.74	-2.89, 1.41	0.50
Fat-free mass (%)	55.3	55.6	53.9, 57.4	56.3	54.7, 57.8	-0.61	-2.29, 1.06	0.47
**Skinfold thickness**								
Biceps area (mm)	21.2	16.4	13.9, 18.9	17.6	15.5, 19.8	-1.21	-3.77, 1.36	0.36
Triceps area (mm)	30.0	26.6	24.0, 29.2	26.9	24.7, 29.1	-0.31	-3.04, 2.42	0.82
Subscapular area (mm)	32.1	27.9	25.1, 30.8	30.2	27.8−32.6	-1.29	-5.335, 0.78	0.14
**Blood pressure**								
Systolic BP (mm/Hg)	124.4	117.0	112.6, 121.4	124.0	120.5, 127.5	-6.98	-12.39, −1.56	0.01
Diastolic BP (mm/Hg)	76.0	73.1	69.8, 76.3	78.4	75.8, 81.0	-5.37	-9.29, -1.44	<0.01
**Blood measurements**								
Fasting glucose (mmol/l)	4.8	5.0	4.8, 5.3	5.1	4.8, 5.3	-0.04	-0.336, 0.29	0.83
120-min glucose (mmol/l)	6.0	5.3	4.6, 6.0	5.9	5.3, 6.5	-0.56	-1.44, 1.32	0.21
Insulin (pmol/l)	134.0	97.9	66.7, 129.2	136.8	112.5, 161.8	-39.31	-75.70, -2.85	0.04
HbA1c (%)	5.2	5.3	5.2, 5.55	5.4	5.3, 5.5	-0.09	-0.27, 0.10	0.35
Insulin C-peptide (nmol/l)	0.6	0.7	0.6, 0.8	0.7	0.6, 0.8	-0.00	-0.12, 0.12	0.99
Triglycerides (mmol/l)	1.4	0.9	0.6, 1.2	1.0	0.8, 1.2	-0.12	-0.47, 0.23	0.51
Ferritin (pmol/l)	116.8	62.0	44.9, 79.3	63.8	50.6, 77.7	-1.71	-23.30, 19.91	0.88
HDL cholesterol (mmol/l)	1.7	1.5	1.3, 1.6	1.5	1.4, 1.6	-0.02	-0.18, 0.14	0.81
LDL cholesterol (mmol/l)	2.9	3.2	2.8, 3.7	3.2	2.8, 3.5	−0.07	-0.47, 0.61	0.80
Total cholesterol (mmol/l)	5.0	5.0	4.5, 5.5	5.1	4.7, 5.5	-0.12	-0.72, 0.48	0.69
Haemoglobin (g/l)	126.6	127.4	122.9, 131.9	129.5	126.0, 130.0	-2.10	-7.60, 3.30	0.44

High-sensitivity CRP (mg/l)	10.7	4.3	2.4, 6.2	4.8	3.3, 6.2	-0.49	-2.86, 1.88	0.69
HOMA2-IR	2.5	3.1	2.2, 4.1	4.9	3.3, 6.5	-1.76	- 3.90, 0.37	0.06

Missing: The number of missing in the exercise and the control group varied between 1 and 3.

Statistics: The effect of treatment was assessed with linear mixed models. For the primary and secondary outcomes, the effect of time and treatment was taken as a fixed effect. Due to randomization, no systematic differences between groups at baseline were assumed.

Abbreviations: PPWR, postpartum weight retention. BMI, Body mass index. BP, blood pressure. HbA1c, Glycated Haemoglobin. HDL, High-density lipoprotein. LDL, Low-density lipoprotein. CRP, C-reactive protein, HOMA2-IR, homeostatic assessment of insulin resistance.

*PPWR[1], postpartum weight minus weight at early pregnancy.

**PPWR[2], postpartum weight minus pre-pregnancy weight. Weight at pre-pregnancy was based on self-reported data. Mean pre-pregnancy weight for all participants were 94.4 kg.

*****Body composition was measured by air displacement plethysmography (BOD POD).
