# Supplementary material for: Exercise training during pregnancy reduces circulating insulin levels in overweight/obese women postpartum: secondary analysis of a randomised controlled trial (the ETIP trial)
Source: BMC Pregnancy Childbirth. 2018 Jan 8;18:18. doi: 10.1186/s12884-017-1653-5 (PMC5759335; doi:10.1186/s12884-017-1653-5)
Supplement: Supplementary file 2 — Per protocol analysis of categorical outcome vairables at three months postpartum. (PDF 161 kb) [file 12884_2017_1653_MOESM2_ESM.pdf]

Additional Table 2. Outcomes at three months postpartum, per protocol. Analysis for the exercise per protocol group and the control group and comparison between groups are presented in number of participants (N), percentage (%), odds ratio (OR), 95% confidence interval (CI), and p-value.

	**Per protocol Exercise group (n = 19)**	**Control group (n = 34)**	**Between-Group Comparison**		
	n (%)	*n (%)*	*Odds Ratio*	*95% CI*	*p - value*
Type 2 diabetes	0 (0)	3 (9.1)	0.63*	0.50, 0.78	0.54
Hypertension	1 (5.6)	3 (10.0)	1.9	0.18, 19.7	1.00
Physical activity ≥ 150 minutes/week**	12 (80.0)	22 (78.6)	0.92	0.19, 4.34	1.00
Exercise training***	7 (46.7)	7 (25.9)	0.40	0.11, 1.51	0.19
Exclusively breastfeeding	8 (50.0)	21 (77.8)	3.50	0.92, 13.3	0.93
Breastfeeding 3-4 meals/24h	3 (18.8)	1 (3.7)	0.17	0.12, 1.76	0.14

Missing: Type 2 diabetes: Exercise group 1 missing, control group 1 missing. Hypertension: Exercise group 1 missing, control group 4 missing. Physical activity questionnaire: Exercise group 4 missing, control group 6 missing. Lactating questionnaire: Exercise group 3 missing, control group 7 missing.

Statistics: The data were analyzed by Fisher's Exact test.

Definitions: Type 2 diabetes: Fasting plasma glucose ≥ 7.0 mmol/l or 2 h concentration ≥ 11.1 mmol/l, according to the definition of the World Health Organization (WHO). Hypertension: Systolic blood pressure ≥ 140, diastolic blood pressure ≥ 90.

* For cohort control group.

**Physical activity ≥ 150 minutes/week: 30 minutes of daily physical activity.

*** Exercise training ≥ 90 min with moderate intensity and/or ≥ 45 min with high intensity per week
